# Small bowel perforation due to CMV enteritis infection in an HIV-positive patient

**DOI:** 10.1186/1756-0500-6-45

**Published:** 2013-02-04

**Authors:** Nick Michalopoulos, Konstantina Triantafillopoulou, Eleni Beretouli, Styliani Laskou, Theodossis S Papavramidis, Ioannis Pliakos, Prodromos Hytiroglou, Spiros T Papavramidis

**Affiliations:** 1 Department of Surgery, AHEPA University Hospital, Aristotle University of Thessaloniki, 85 Karakasi Str, Thessaloniki, Greece; 2 Department of Pathology, AHEPA University Hospital, Aristotle University of Thessaloniki, Thessaloniki, Greece

**Keywords:** CMV enteritis, Small bowel perforation, HIV infection

## Abstract

**Background:**

Cytomegalovirus infection of the gastrointestinal tract is common and is more often seen in patients with acquired immunodeficiency syndrome (AIDS). Although small bowel infection is less common than infection of other parts of the gastrointestinal system, it may lead to perforation, an acute complication, with dreadful results.

**Case presentation:**

This article reports a case of Cytomegalovirus ileitis with multiple small bowel perforations in a young man with human immunodeficiency virus (HIV) infection. The patient developed abdominal pain with diarrhea and fever, and eventually acute abdomen with pneumoperitoneum. The patient had poor prognosis and deceased despite the prompt surgical intervention and the antiviral therapy he received. At pathology a remarkable finding was the presence of viral inclusions in smooth muscle fibers. The destruction of muscle cells was the main cause of perforation.

**Conclusion:**

Morbidity and mortality associated with perforation from CMV enteritis in AIDS patients are high and the life expectancy is short. Cytomegalovirus disease is multifocal; therefore, excision of one portion of the gastrointestinal tract may be followed by a complication elsewhere. Our case elucidate that muscle cell destruction by the virus is a significant cause leading to perforation.

## Background

Cytomegalovirus (CMV) is a well-recognized pathogen in the general population. It is a DNA virus and a member of the herpes virus group [1,2]. In the normal host, primary infection is usually subclinical. When symptoms are present, they are similar to the syndrome of infectious mononucleosis [3]. After primary infection, CMV, like other herpes viruses, remain latent within the host and can be reactivated later during life [1].

On the contrary, CMV significant disease, either primary or reactivated is typically seen in immunocompromised individuals such as chemotherapy, transplant and acquired immunodeficiency syndrome (AIDS) patients [1,3,4]. In these hosts CMV disease usually presents with specific organ involvement like retina, respiratory system, central nervous system or gastrointestinal (GI) tract.

CMV infection of GI tract is common in patients with AIDS [5]. The GI tract may be affected anywhere from the mouth to the anus. The most common site, though, is the colon (47%), followed by the duodenum (21.7%), stomach (17.4%), esophagus (8.7%), and rarely small bowel (4.3%) [6]. Patients with advanced HIV infection, particularly if the CD4 count less than 50cell/IU, are at high risk to develop a life-threatening complication following CMV enteritis. Bleeding of GI tract and perforation of the colon are more commonly seen [7,8]. Perforations of the small intestine are rarely encountered after CMV enteritis in patients with AIDS [9,10]. We report a case of multiple small bowel perforations due to CMV infection in an immune-suppressed patient with AIDS. We emphasized in the pathogenesis of perforation and we review the literature on the clinical presentation, diagnosis, management and outcome of CMV infection in HIV-positive patients.

## Case presentation

A 29-year-old Caucasian, Greek seropositive man with HIV infection was admitted to the emergency department of our hospital complaining for fever, cough and dyspnea. He was also suffering from nausea, vomiting, loss of appetite and abdominal pain. HIV infection had been diagnosed 12 months previously and the patient was under highly active antiretroviral therapy (HAART). HAART included emtricitabine/tenofovir disoproxil fumarate (Truvada) and saquinavir (Invirase). An upper respiratory tract infection was diagnosed and the patient was hospitalized. Tuberculosis (TB) infection was suspected and though the tests for TB proved to be negative the patient was treated with moxifloxacin and also received anti-TB therapy. Two months previous to this presentation, the patient was hospitalized for melena. Then, colonoscopy showed chronic ileitis, possibly due to Crohn’s disease or CMV infection. However, CMV serum and stool tests were negative. The abdominal computed tomography (CT) revealed no specific findings. After ten days of pharmaceutical treatment with moxifloxacin and anti-TB dugs the infection regressed and the patient was discharged.

**Figure 1:**
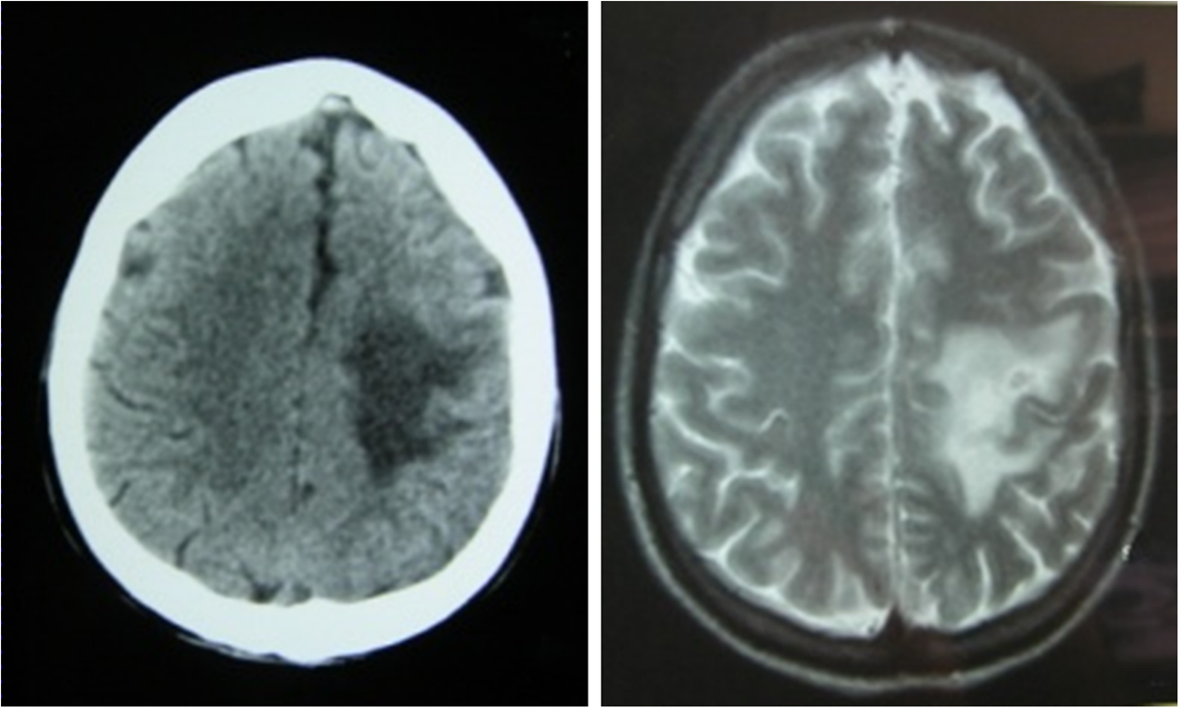
Cerebral MRI showing enhanced lesion in the left lobe.

Nevertheless, 2 months later he was referred again to our hospital with more severe respiratory and gastrointestinal symptoms including fever (>39°C), cough, dyspnea, dizziness, nausea, vomiting, abdominal pain and diarrhea. At the second hospitalization HIV levels were extremely high (528571 cop/ml) and CD4 lymphocytes were less than 100cell/IU. Additionally to chest X-rays images, a CT scan of thorax was performed and showed no specific findings. Moreover, the patient presented an acute neurological right-sided syndrome with hemiparesis, positive Barre sign, positive Babinski sign, and central prosopoplegia. A cerebral magnetic resonance image (MRI) subsequently was performed and showed enriched focal lesions with edema in the left parietal lobe and near the medial frontal cornu. Similar lesions, but without enrichment, were also present in the right temporal area near the left frontal cornu (Figure 1). The differential diagnosis included tuberculosis, fungal infection, toxoplasmosis and lymphoma. A prompt anti-toxoplasma pharmaceutical treatment was begun and the neurological symptoms seemed to subside, although the new MRI images had not improved.

**Figure 2:**
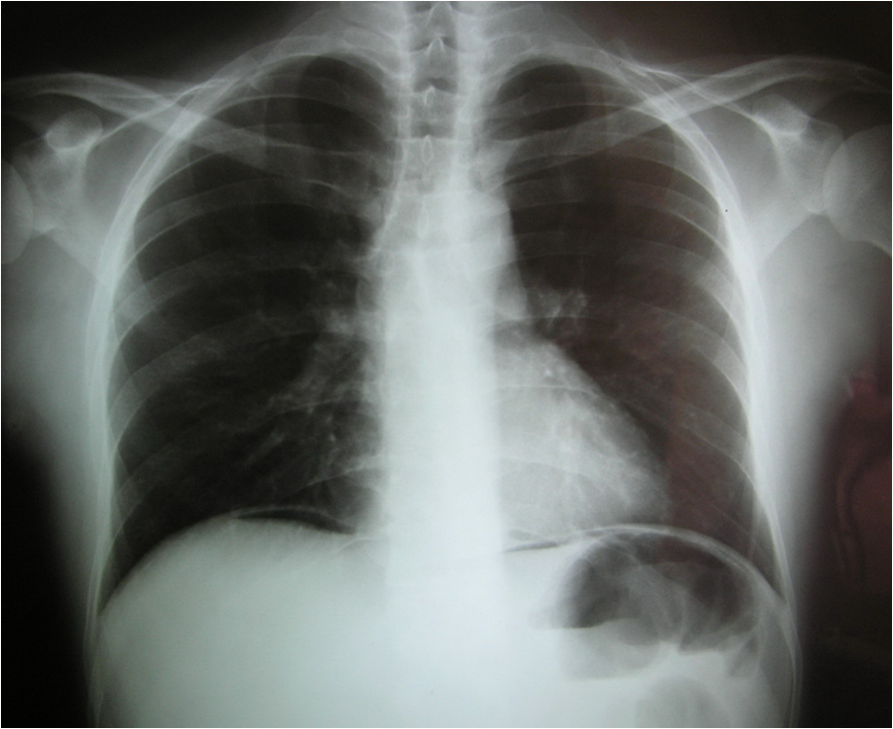
Chest X-ray revealing pneumoperitoneum.

Ten days later the patient complained for progressive abdominal pain. During the physical examination, rebound tenderness was present. Chest X-ray was indicative of pneumoperitoneum (Figure 2). The patient was prepared for emergency surgical intervention. At laparotomy, two perforated small-bowel ulcers were detected, one near the ileocolic valve and the other one near the ligament of Treitz. Moreover, multiple discolorations on the serosa surface of small intestine were presented. After biopsy specimens were taken, the ulcers were sutured and drains were placed. Also, a biopsy was taken from the liver. On the 3^rd^ postoperative day the patients’ condition was deteriorated. He complained of progressive thoracic and abdominal pain, while being dyspneic and tachycardic. CT images revealed bilateral pleural, parasplenic and left paracolic effusions, as well as small bowel dilatation. Simultaneously, stercoraceous fluid filled the drainage bag and the patient was led to the operating theater for an emergency re-laparotomy. Multiple perforations were found in the small bowel. Partial enterectomy was performed and peritoneal cavity was exhaustively irrigated using sterile isotonic sodium chloride solution. New drains were placed, the abdominal wall was closed and re-laparotomy was decided to perform on demand. The patient was transferred to the intensive care unit and stabilized. During the postoperative course he seemed to be in a stable condition until the sixth postoperative day, when he became septic again. The abdominal trauma was found suppurated, while new pleural and abdominal effusions were detected on abdominal CT scans. Moreover, cerebral CT scans revealed intense edema and two edematous lesions by the basal ganglia. Despite aggressive antibiotic and anti-edema treatment, the patient rapidly deteriorated and deceased due to cerebral intussusception.

**Figure 3:**
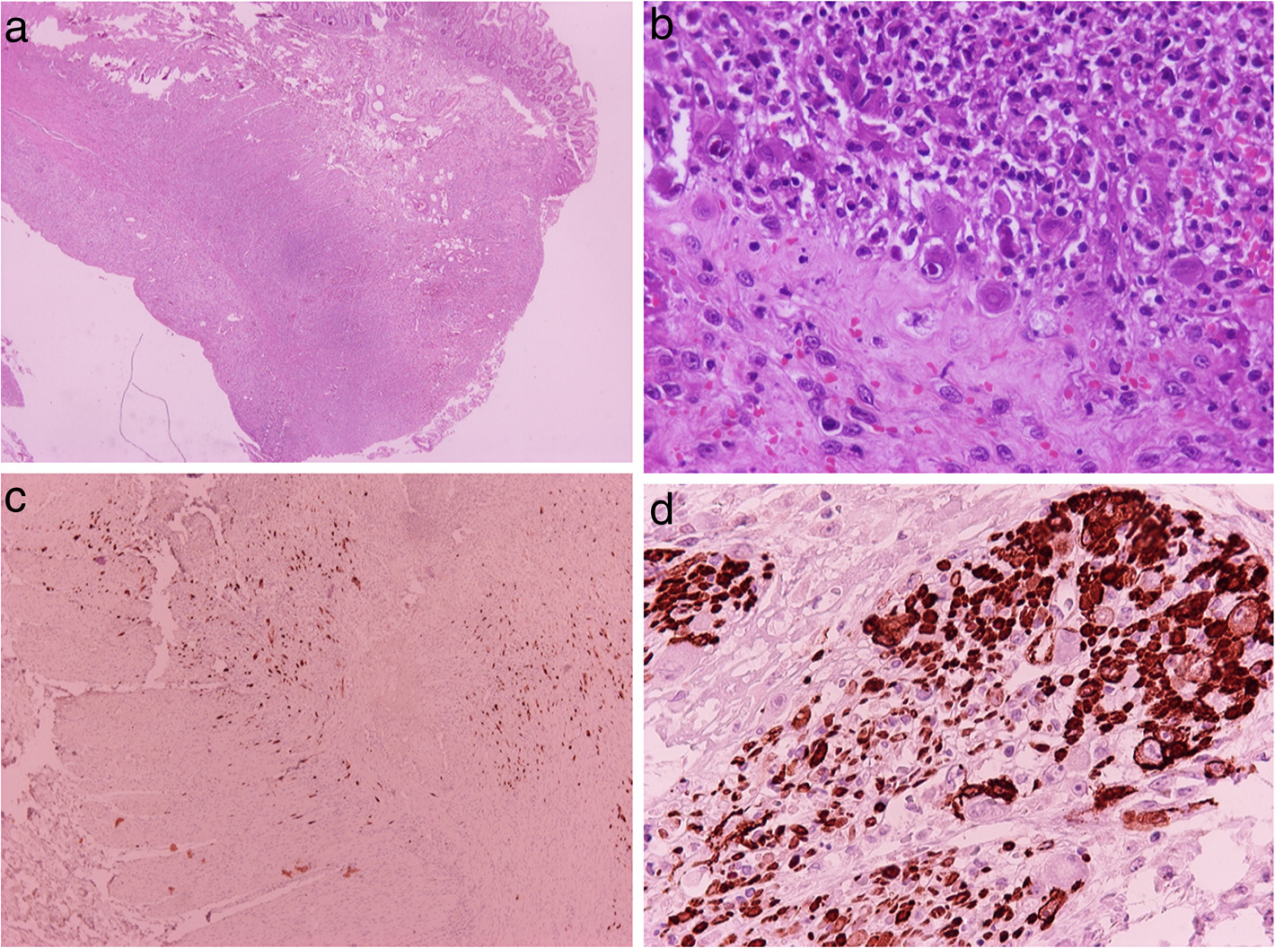
**Histologic and immunohistochemical findings.** Footnote: **a**: H&E X 20. Perforation site of small bowel with necrosis and acute and chronic inflammation. **b**: H&E X 400. Enlarged cells 90^o^ 2/3 contrast with typical CMV inclusions in ulcer. **c**: Streptavidin-Biotin X 100. A large number of cells are positive for CMV early antigen. **d**: Streptavidin-Biotin X 400. CMV inclusions are readily identified in cells of the muscularis propria on stain for smooth muscle actin.

Histologic examination of the ileal biopsies and resection specimens showed multiple areas of mucosal ulceration with acute and chronic inflammation. Transmural inflammation and necrosis was found at the perforation sites (Figure 3a). A large number of cells were enlarged and contained typical CMV inclusions (Figure 3b). Most of these infected cells were endothelial, but other cells types, such as smooth muscle fibers, also contained viral inclusions, as confirmed by immunohistochemical stains (Figure 3c,d). Histologic examination of the liver biopsy showed scattered aggregates of inflammatory cells and occasional large cells with characteristic CMV inclusions. Small numbers of positive cells were seen on immunostains for CMV early antigen.

## Discussion

CMV gastrointestinal tract infection typically presents with anorexia, fever, diarrhea, abdominal pain, wasting, weight loss and is associated with a wide range of lesions, ranging from mild inflammation and erosions to esophageal and enterocolic ulcers [11-13]. The incidence of CMV gastrointestinal disease and the related complications was rapidly increased in HIV-positive individuals and currently are probably greater than apreciated [8,12]. CMV has been reported to be the most common cause of lower GI bleeding in AIDS patients and bleeding without diarrhea may be the initial manifestation of CMV colitis [14]. Bleeding may result from severe pan-colitis, segmental colitis or isolated well circumscribed ulcers [15]. Perforation is the most lethal complication and is commonly seen between ileum and splenic flexure [8,16]. Other rare complications are toxic megacolon [17] and acute appendicitis [16].

The pathogenesis of CMV related GI disease is believed to be submucosal vasculitis resulting in thrombosis with ischemia leading to ulceration, bowel wall thickening and occasional gangrene or perforation [18]. Specifically, two mechanisms by which CMV could be a factor leading to perforation are proposed by Goodman et al. [19]; It is possible that viral infection of the colonic epithelium would result in mucosal erosion and ulceration, secondary infection of the granulation tissue and further ulceration, leading eventually to perforation. An alternative mechanism might be CMV infection of granulation tissue in a preexisting ulcer which might otherwise have healed, causing exacerbation of the inflammation, further ulceration, and eventually perforation. A remarkable finding of our case was the presence of extensive CMV inclusions in fibers of the muscularis propria, apparently resulting in muscle cell necrosis and perforation. Muscle cell destruction has been suggested as the basis for perforation by Fernandes et al. [20] and was further supported by the findings in the studies of Francis et al. [21] and Genta et al. [13]. Francis et al. [21] found CMV inclusions in muscolaris mucosa cells especially in the colon. They proposed that the infection of muscles cells is made by infected pericytes, although no intestinal perforation was observed in their series [21]. The findings in our case strongly support the theory of muscle cell destruction.

The appearance of the perforated intestine due to CMV infection in patients with AIDS is characteristic and usually reveals multiple mucosal ulcerations with one or more full-thickness perforations through an ulcer base; multiple brownish discolorations on the serosal surface that corresponded in locations where the mucosal ulcerations are presented [8,22]. The endoscopic patterns of CMV colitis in AIDS are heterogenous, although subepithelial hemorrhage, colitis, and ulcers are typical. The endoscopic appearance could mimic pseudomembranous colitis, ulcerative colitis or Crohn’s disease [22,23].

The diagnosis of CMV infection is made commonly by pathology results, especially in cases where the lesions may appear macroscopically normal [24,25]. The microscopic examination of these ulcers characteristically reveals inflammation and granulation tissue containing large cells with nuclei showing the typical CMV inclusions [8,26]. CMV inclusion cells indicate active virus production and they are usually associated with pathological lesions [21]. Diagnosis is further facilitated by immunohistochemistry, in situ hybridization and polymerase chain reaction [13,21]. Serology is not sufficient to make a timely diagnosis of CMV infection, and the absence of CMV IgM antibody may not exclude CMV infection. In HIV antibody positive patients the underlying immune deficiency makes serological diagnosis of active CMV infection unreliable [21]. In our case the initial serology tests were negative and not indicative of CMV infection. Therefore PCR should be performed for CMV involvement in HIV positive patients, particularly when CD4 levels are low.

Cytomegalovirus ileocolitis is responsible for the majority of emergency laparotomies in AIDS patients [9]. Thus, the clinical entity of small bowel perforation due to CMV infection needs to be recognized and may be associated with the development of acute abdominal pain in the setting of longstanding pain, chronic diarrhea, and fever [27]. Emergent surgical intervention is mandatory to these patients. Because of the multifocal nature of CMV, distal small-bowel perforations should probably be treated by segmental resection with an end-stoma and mucous fistula, whereas right colectomy is also acceptable [9]. Nevertheless, there is a high rate of perforation in other parts of the bowel on the postoperative period [16]. Overall, CMV ileocolitis is directly responsible for the deaths of 54% to 87% of all AIDS patients who underwent emergent laparotomy [8,9]. The uncomplicated CMV enteritis should be treated conservative with ganciclovir which has been used increasingly in the therapy of CMV disease [28,29]. The administration of ganciclovir is effective as first-line treatment for gastrointestinal CMV infection, but maintenance therapy does not prevent the progression of disease [28,29]. If ganciclovir cannot be tolerated, or if resistance develops against it, foscarnet and cidofovir are available as intravenous drugs [11,29]. Another therapeutic strategy proposed by Soderlund et al. [12] is the elective surgical resection of the inflamed intestine, prior the formation of perforation, in combination with anti-CMV therapy which seems to offer good palliation and survival.

## Conclusions

Morbidity and mortality associated with perforation from CMV enteritis in AIDS patients are high and the life expectancy is short. Cytomegalovirus disease is multifocal; therefore, excision of one portion of the gastrointestinal tract may be followed by a complication elsewhere. Last but not least, the importance of histologic examination in reaching a timely diagnosis in these patients is emphasized. Our case elucidate that muscle cell destruction by the virus is a significant cause leading to perforation.

## Consent

Written informed consent was obtained from the patient’s closest relatives for publication of this Case report and any accompanying images. A copy of the written consent is available for review by the Series Editor of this journal.

## Abbreviations

AIDS: Acquired immunodeficiency syndrome; HIV: Human immunodeficiency virus; CMV: Cytomegalovirus; GI: Gastrointestinal; HAART: Highly active antiretroviral therapy; TB: Tuberculosis; CT: Computed tomography; MRI: Magnetic resonance image.

## Competing interests

The authors declare that they have no competing interests.

## Authors’ contributions

NM received the patient in our out-patient department, was the principal surgeon and drafted the manuscript. KT revised the manuscript critically for important intellectual content. EM performed the pathological examination and was a major contributor in writing the manuscript. SL analyzed and interpreted the patient data and drafted the manuscript. TP was an auxiliary surgeon and had significant contribution to conception and design of the manuscript. IP was an auxiliary surgeon was a major contributor in writing the manuscript. PX performed the pathological examination and immunohistochemistry and revised critically the manuscript. SP was responsible for the overall treatment of the patient, revised critically the manuscript and has given final approval of the version to be published. All authors read and approved the final manuscript.
